# Differentiating Induced Pluripotent Stem Cells into Renal Cells: A New Approach to Treat Kidney Diseases

**DOI:** 10.1155/2020/8894590

**Published:** 2020-08-07

**Authors:** Patrícia de Carvalho Ribeiro, Lucas Felipe Oliveira, Mario Abbud Filho, Heloisa Cristina Caldas

**Affiliations:** ^1^Laboratory of Immunology and Experimental Transplantation-LITEX, Medical School of Sao Jose do Rio Preto, Sao Jose do Rio Preto, Sao Paulo, Brazil; ^2^Physiology Division, Natural and Biological Sciences Institute, Triangulo Mineiro Federal University, Uberaba, Minas Gerais, Brazil; ^3^National Institute of Science and Technology for Regenerative Medicine, Rio de Janeiro, Rio de Janeiro, Brazil; ^4^Kidney Transplant Unit, Hospital de Base, FAMERP/FUNFARME, Sao Jose do Rio Preto, Sao Paulo, Brazil; ^5^Urology and Nephrology Institute, Sao Jose Rio Preto, Sao Paulo, Brazil

## Abstract

Renal disease is a major issue for global public health. Despite some progress in supportive care, the mortality rates among patients with this condition remain alarmingly high. Studies in pursuit of innovative strategies to treat renal diseases, especially stimulating kidney regeneration, have been developed. In this field, stem cell-based therapy has been a promising area. Induced pluripotent stem cell-derived renal cells (iPSC-RCs) represent an interesting source of cells for treating kidney diseases. Advances in regenerative medicine using iPSC-RCs and their application to the kidney are discussed in this review. Furthermore, the way differentiation protocols of induced pluripotent stem cells into renal cells may also be applied for the generation of kidney organoids is also described, contributing to studies in renal development, kidney diseases, and drug toxicity tests. The translation of the differentiation methodologies into animal model studies and the safety and feasibility of renal differentiated cells as a treatment for kidney injury are also highlighted. Although only few studies were published in this field, the results seem promising and support the use of iPSC-RCs as a potential therapy in the future.

## 1. Introduction

Kidney disease is a condition characterized by impaired renal function and/or structure [1, 2]. Its incidence has increased over the years and represents a considerable concern worldwide [3, 4]. Kidney diseases can be distinguished into acute kidney injury (AKI) and chronic kidney disease (CKD), although intercommunication between these two pathologies has been observed [5].

AKI is characterized by a rapid decline in renal function and excessive renal inflammation, as well as programmed death of resident cells [6–8]. In addition, AKI shows high morbidity and mortality and may progress to CKD [6]. Conversely, CKD is defined as the irreversible impairment of renal function and/or structure for 3 months or more [9] and its major causes are systemic arterial hypertension and diabetes [10]. Both AKI and CKD may progress to end-stage renal disease (ESRD), a condition with very few effective and specific available therapies, except for supportive care [11]. ESRD reduces quality of life in patients, significantly diminishes life expectancy, and increases health care costs [12].

The high incidence of renal diseases has caused a relentless pursuit of effective therapeutic methods, aiming to slow down or even stop the progress of the disease. Several strategies have been developed over the time, including the first attempt to create an artificial kidney in the 1940s [13], the long-term successful human kidney transplantation from a living donor [14], the introduction of outpatient dialysis in the 1960s [15, 16], and the discovery of drugs that delay the progression of kidney disease, such as the renin-angiotensin-aldosterone system blockers [17]. Nevertheless, further strategies that effectively and ideally remove patients from the transplant queue are still needed. Therefore, the development of new therapeutic strategies is crucial and cellular therapy has emerged as a promising field to achieve this goal.

Adult renal tissue has a limited regeneration capacity after an injury [18]. In this context, there is growing interest in the study of regenerative cell therapy in kidney diseases, especially those involving the use of renal cells derived from induced pluripotent stem cells (iPSC). Since iPSC are immature cells [19] and can originate almost any cell type in the body, differentiation protocols commonly attempt to mimic the embryonic development of the kidneys [20]. Unlike pluripotent stem cells, renal cells have a limited number of divisions and are at a more mature stage of differentiation, representing a safer option for cell therapy [21].

Potential applications of iPSC-RCs are described in the present review, as well as discussions on the advances in regenerative medicine and the safety and feasibility of renal differentiated cells as a treatment for kidney injury.

## 2. Embryonic Development of the Mammalian Kidney

Understanding kidney organogenesis is important to establish a wide range of cell differentiation methodologies. The mammalian kidney originates from the intermediate mesoderm (IM) by the sequential induction of three distinct kidneys: pronephros, mesonephros, and metanephros [22, 23]. During the development process, these structures receive various inductive signals and interactions from the environment in order to become kidneys [22]. The expression of transcription factors PAX2, PAX8, and LHX1 is common to all of them [24, 25]. The first structure to arise is the pronephros, followed by the mesonephros, both degenerating before birth [23]. However, the metanephros is the last to arise and the only one to persist and form the permanent organ with all its individual functional units—the nephrons [22, 23, 26].

In the adult kidney, nephrons are originated through reciprocal signal induction between two IM structures: ureteric bud (UB) and metanephric mesenchyme (MM) [22]. The UB is an epithelial side branch of the Wolffian duct [27], and after induction by glial cell line-derived neurotrophic factor (GDNF), produced by the MM, it evolves towards the MM initiating a series of dichotomous branching and leading to the ureteric epithelial tree development, which in turn will originate the collecting ducts in the metanephros [26–28]. At this stage, the GDNF is continued produced by a specific mesenchymal cell population, named cap mesenchyme, which represents nephron progenitor cells (NPCs) and expresses SIX2 transcription factor [29]. The expression of SIX2 is essential for maintaining the NPC in an immature stage, and its cessation is related to the initiation of nephron commitment [30]. Increased levels of the canonical Wnt9b signaling [31], as well as the Notch signaling [30], have been suggested as inductive of a mesenchyme-to-epithelial transition (MET), initiating the differentiation into nephron cells [27, 30].

Subsequently, a pretubular aggregate of mesenchyme gives rise to a renal vesicle, which develops a lumen and grows towards the distal end of the ureteric tip to form a contiguous lumen with the ureteric epithelium, enabling the appropriate drainage from the nephron through the collecting ducts [32]. The renal vesicle then elongates into a comma-shaped body that undergoes further morphological alteration into S-shaped body [30]. Following that, a glomerulus formation is initiated with a capillary loop invasion into a region denominated glomerular cleft, located between the primitive podocytes and the proximal tubule, in the S-shaped body [30, 33, 34]. During the glomerular maturation, the capillary is divided in several loops, endothelial cells became fenestrated, all the capillary structure is enveloped by the glomerular basement membrane, and podocytes extend their foot processes around the endothelial cells [34]. Some of the transcription factors expressed in early stages of podocytes maturation are LMX1B, FOXC2, POD1, FOXD2, and MAFB [35–39].

**Figure 1:**
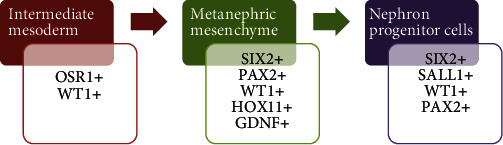
Kidney development stages and expression of transcription factors. OSR1 and WT1 are expressed in the intermediate mesoderm; SIX2, PAX2, WT1, HOX11, and GDNF are expressed in the metanephric mesenchyme; coexpression of SIX2, SALL1, WT1, and PAX2 characterizes a NPC.

Regarding other renal development markers, PAX2 and WT1 are expressed at the beginning of the kidney rising and then downregulated [40]. However, they become active again at the final stages of nephron formation. Furthermore, OSR1 transcription factor is expressed in the intermediate mesoderm, while HOX11 is expressed in the metanephric mesenchyme and the coexpression of SIX2, SALL1, WT1, and PAX2 characterizes a NPC [22] (Figure 1).

## 3. Pluripotent Stem Cell for Cell-Based Therapy

Over the past two decades, we have experienced growing interest in the use of stem cells as a therapeutic alternative for regenerating damaged tissues and organs. Stem cells are characterized by a large proliferative ability and potential to differentiate into distinct specialized cells. It is also noteworthy that not all stem cell types possess the same differentiation and therapeutic potentials, since pluripotent stem cells exhibit higher potential than multipotent ones [41].

Pluripotent stem cells are self-renewing, clonogenic, and able to undergo lineage commitment into the three different embryonic germ lines: ectoderm, mesoderm, and endoderm [42]. The most famous source of these cells is human embryos at blastocyst phase, namely, embryonic stem cells (ESCs). However, the use of ESC for cellular therapy is quite complex, considering ethical conflicts concerning manipulation of human embryos and safety concerns related to their immunogenicity, as well as the risk of uncontrolled growth and teratoma formation when administrated in vivo [43].

In an attempt to overcome these issues, a new reprogramming technology has led to the generation of iPSC from somatic cells through the introduction of four factors: Oct4, Sox2, c-Myc, and Klf4 [19]. iPSC share with ESCs many features including pluripotency and high differentiation capacity, representing a promising alternative as a source of pluripotent stem cells without ethical concerns and immunorejection, since they can be generated from patient-derived adult cells [41].

Although the application of iPSC in regenerative medicine seems to be promising, their use *per se* in cellular therapy is challenging. Some limitations still persist and include the efficiency of their derivation, the risk of tumor development following transplantation due to their high proliferative potential [21, 44], and the use of viral vectors for reprogramming [45], restricting the iPSC application in an immature stage. Therefore, an alternative approach is to differentiate iPSC into a specific cell type before cellular transplantation. Such differentiation protocols enable the management of crucial variables for cell therapy, some of which are cell fate and expansion in culture.

## 4. iPSC-Derived Renal Cells

Until recently, renal studies were made only with immortalized kidney cell lines or animal model systems [46]. However, immortalized kidney cells obtained from primary cultures have some limitations, including complications for successful isolation, short-time life periods in culture, and restricted functional and/or morphological characteristics when compared to their native counterparts [47, 48]. Since the iPSC advent [19], great interest has arisen in studying these cells for several diseases and drug development models. A major advantage in the use of iPSC is that they can be generated from somatic cells, enabling immunocompatible transplantation and development of patient-specific models of disease [44].

In vitro differentiation of iPSC into kidney cells can be achieved by the induction of specific nephrogenic factors. In general, a common step in the differentiation protocols is the use, among other substances, of at least two of these three nephrogenic factors: activin A, retinoic acid (RA), and bone morphogenetic proteins (BMPs). These factors have an important role in the generation of kidney structures and specification of renal progenitor cells during renal development. The use of activin A and RA has been described as capable of generating structures related to kidney development [49, 50]. The ureteric bud produces activin A during the kidney growth phase, and it is an important nephrogenic factor, inducing the differentiation into metanephric mesenchyme [51]. Similarly, RA is a crucial factor during kidney development and the specification of renal progenitor cells [52]. The blockage of RA action in this phase causes serious abnormalities to the urinary system [53]. BMP7 also plays an important role in the kidney formation, and its genetic ablation results in highly disorganized and undeveloped kidneys, with an expanded interstitium [54]. In this context, diverse differentiation methodologies have been described in the past few years, aiming to transform iPSC into renal cells with similar properties to those observed in vivo (Table 1).

### 4.1. Differentiation Protocols

Kim and Dressler were the first to use a combination of activin A, BMP7, and RA to differentiate pluripotent stem cells into renal cells [55]. They induced embryoid body (EB) formation and then differentiated mouse embryonic stem cells (ESC) into cells expressing markers for intermediate mesoderm and early derivatives of the metanephric mesenchyme, such as PAX2, WT1, LIM1, GDNF, Cadherin-6, and EYA1. In addition, they injected the resulting cells into a developing kidney and observed their integration into tubules, along with the expression of proximal tubule markers.

Following this work, Morizane et al. [56] have used iPSC for the generation of kidney cells, which expressed SIX2, WT1, PAX2, Nephrin, and KSP (the last one being a tubular specific marker). The authors generated iPSC from mouse fibroblasts and then initiated the differentiation by the induction of embryoid body (EB) formation, followed by cell plating in gelatin-coated dishes. During the entire process, activin, GDNF, and BMP7 or only activin was added to the differentiation media. When the three nephrogenic factors were used, the authors found that the iPSC could differentiate into metanephric mesenchyme cells, while the sole use of activin enables the generation of tubular cells.

In 2012, Song and collaborators described the direct differentiation into renal cells using human iPSC [57]. The iPSC were generated from normal human kidney mesangial cells and induced to differentiate into renal progenitor cells (RPCs). Activin A, BMP7, and RA were used as nephrogenic factors. The protocol was initiated with the EB formation, followed by adherent culture, for 10 days. At the end of the protocol, cells were characterized and they showed the expression of Nephrin, Synaptopodin, PAX2, and WT1, as well as functional properties similar to those observed in podocytes from primary culture. Furthermore, the cells were able to proliferate in vitro and could be maintained up to 3 months. Later, several new studies were published reporting the generation of different types of renal cells and improving the differentiation protocols [58–84] available.

### 4.2. Kidney Organoids

The evolution on the knowledge related to kidney organogenesis enabled the creation of enhanced methodologies, in special the ones involving 3D mini-organs, the organoids, which host several kinds of renal cells [60]. Takasato et al. [68, 69] have developed a 3D differentiation protocol by which kidney organoids were generated. Using CHIR, FGF9, and heparin in a series of methodological steps for 25 days, the authors described the formation of a 3D structure, which consisted of multiple nephron segment cells, expressing markers for glomerulus (WT1^+^ cells), early distal tubule (GATA3^−^ LTL^−^ ECAD^+^ cells), early proximal tubule (LTL^+^ ECAD^−^ cells), and collecting duct (GATA3^+^ ECAD1^+^ cells). Renal structures observed during the differentiation protocol resembled in vivo kidney tissue organization, and each organoid comprised a substantial size with more than 500 nephrons.

Morizane and collaborators [70] also described a kidney organoid generation, mainly through CHIR and FGF9 induction in a 3D culture. The authors first differentiated human iPSC into primitive streak cells, following induction into posterior intermediate mesoderm and nephron progenitor cells. These cells were transferred to a 3D culture and treated with CHIR and FGF9 and by day 21; the renal organoids were spontaneously organized in elongated epithelial nephron structures expressing several nephron markers. These structures expressed nephron markers in a contiguous arrangement, including loops of Henle (E-cadherin (CDH1)^+^ uromodulin (UMOD)^+^ BRN1^+^ AQP1^+^), distal convoluted tubules (CDH1^+^UMOD^−^), glomerular podocytes (NPHS1^+^PODXL^+^WT1^+^), and proximal tubules (LTL^+^AQP1^+^).

Over the past few years, several other protocols involving kidney organoid generation [71, 75, 76, 82] were also described, enabling the use of such differentiated cells for experimental models in kidney disease.

## 5. iPSC-Derived Renal Cells as Cell Therapy for Kidney Diseases

The ability to self-renew and differentiate makes stem cells a promising strategy for regenerating damaged kidneys. Our group has recently published a work [44] studying the therapeutic potential of iPSC in a CKD model in rats (the 5/6 model). Although iPSC ameliorated CKD rats, they also generated Wilms' tumors, justifying the essential step of differentiating iPSC into renal cells prior to their transplantation into kidney disease models [21, 44].

Few reports have addressed the regenerative potential of iPSC-derived renal cells in kidney diseases (Table 2). Imberti et al. [64] described the generation of renal progenitor cells from human iPSC and studied their therapeutic potential in a mouse model of AKI. Intravenously infused RPCs integrated into mouse renal tissue as early as 24 h after transplantation, especially into tubuli. Results showed a reduction of blood urea nitrogen (BUN) levels and improved renal histology in mice when compared to the control group.

Toyohara et al. [65] have injected OSR1^+^SIX2^+^ RPCs into the renal subcapsule of induced AKI mice and observed that although the cells did not differentiate into tubular structures, kidney function was improved in the treated animals. In addition, histological analysis demonstrated a significant reduction in renal parenchyma damage. Similarly, Li et al. [66] have transplanted RPCs into an ischemia/reperfusion-induced AKI model in rats and observed improved renal function and histological aspects in the treated group.

More recently, Hoshina et al. [83] studied hiPSC-derived RPCs (CD9^−^CD140a^+^CD140b^+^CD271^+^ cells) as a therapy for AKI. Cells were injected into renal subcapsules after the induction of AKI in a mouse model. The authors described improved renal function and reduced tissue damage, indicated by decreased fibrosis, tubular dilatation, and loss of tubular borders. Ahmadi et al. [81] also studied the potential of renal cells in kidney disease, specifically using iPSC-derived podocytes in a mouse model of membranous nephropathy. As early as 10 days after the cell transplantation, proteinuria was significantly decreased in the treated animals and there was also reduction in the urine albumin/creatinine ratio, indicating the benefits of using mature renal cells (iPSC-podocyte) as cell therapy.

## 6. Future Perspectives

**Figure 2:**
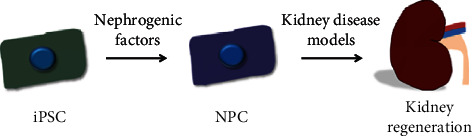
iPSC-derived renal cells in kidney diseases. iPSC: induced pluripotent stem cells; NPC: nephron progenitor cell.

A nephron is a complex structure, composed of multiple varieties of cells [84]. Therefore, addressing which one should be transplanted for treating specific kidney injuries remains a challenge. However, it is expected that the transplantation of kidney progenitors enables the final cell differentiation into the tissue and provides a source of several types of cells, which can be used for renal regeneration and improvement of kidney function [85] (Figure 2).

iPSC represent a valuable choice for cell therapy, considering their ability to generate renal cells at their more primitive lineage stage. Such cells may then be employed for therapeutic proposes, differentiated into a mature cell, or even be used for repopulating decellularized native kidney [86, 87]. The advance in the understanding of the kidney development has provided the refinement of differentiation methodologies leading to improved cost-effective protocols and generation of more types of cells and even more complex and organized structures [88, 89]. 3D conformation culture, associated with specific growth factors, is aimed at mimicking the developmental stages and provides generation of organoids, with a wide range of cell types that are also self-organized in organ-specific structures, resembling their native counterparts [90].

The development of kidney organoids allows their use for regenerative medicine as a source of several types of renal cells (from RPCs to mature podocytes or tubular cells), which could be applied for cell therapy [91]. In addition, such organoids may be used for studying renal embryonic development and diseases, as well as for testing drug toxicity and, therefore, providing a valuable tool for improving in vitro scale, structure, and functional maturation of the kidney in the future [92–94].

Although the studies underlying the use of iPSC-derived renal cells in kidney diseases have promising results, only a few were published and further investigation on whether these cells could effectively be applicable as a treatment or not is needed. Studies in this direction may provide a better understanding of the action mechanisms of renal cells in kidney diseases and their efficacy and safety, as well as the possibility to translate these discoveries from bench to bedside. Further studies are necessary to address the use of iPSC-derived renal cells in CKD. Such cells may represent a promising strategy to slow down the progression of disease and regenerate the damaged tissue.

## 7. Conclusions

The development of innovative iPSC differentiation protocols into renal cells and the advanced knowledge in kidney development enable the emergence of new studies focused on the treatment of kidney diseases. Such studies demonstrate the therapeutic potential of differentiated renal cells, supporting their promising use as cell therapy. Long-term studies are necessary to address the beneficial effects and safety of iPSC-derived renal cells.

## Figures and Tables

**Table 1:** Protocols for iPSC differentiation into renal cells.

Authors	Differentiation factors	Differentiation period	Starting iPSC type	Induced cell type
Morizane et al. 2009 [56]	Activin A, GDNF, BMP7	14 days or 18 days	Murine iPSC	Tubular cells, metanephric mesenchyme cells
Song et al. 2012 [57]	Activin A, BMP7, and RA	10 days	Human iPSC	Podocyte-like cells
Mae et al. 2013 [58]	CHIR99021, Activin A, and BMP7	~10 to 20 days	OSR1-GFP human iPSC	Intermediate mesoderm cells
Xia et al. 2013 [59]	BMP4, FGF2, RA, activin A, and BMP2	4 days	Human iPSC	Ureteric bud kidney progenitor-like cells
Taguchi et al. 2014 [60]	BMP4, activin A, basic FGF, CHIR RA, and FGF9	14 days	Human iPSC	Kidney organoid—Metanephric nephron progenitors
Araoka et al. 2014 [61]	CHIR99021 and AM580 or TTNPB	5 days	Human iPSC and OSR1-GFP human iPSC	Intermediate mesoderm cells
Lam et al. 2014 [62]	CHIR99021, FGF2, RA, FGF9, and activin A	9 days	Human iPSC	Intermediate mesoderm cells
Kang & Han 2014 [63]	Activin A, Wnt3a, BMP4, FGF2, RA, BMP7	26 days	Human iPSC	Nephron progenitor cells
Imberti et al. 2015 [64]	RA, RhoA inhibitor and PI3K inhibitor, activin A, FGF2, BMP7, and GDNF	19 days	Human iPSC	Renal progenitor cells
Toyohara et al. 2015 [65]	Activin A, CHIR, BMP7, TTNPB, TGF-*β*1, and DMH1	28 days	Human iPSC	Renal progenitor cells
Li et al. 2015 [66]	RA, BMP7, activin A, renal epithelial cell growth medium alone	10 days	Mouse iPSC	Renal progenitor cells
Kandasamy et al. 2015 [67]	Renal epithelial growth medium, Rho kinase, BMP2, and BMP7	8 days	Human iPSC	Proximal tubular-like cells
Takasato et al. 2015 [68, 69]	CHIR, FGF9, heparin	25 days	Human iPSC	Kidney organoid–nephron segment cells
Morizane et al. 2015 [70]	FGF2, CHIR, Noggin, activin A, and FGF9	9 days (NPCs) 21-35 days (organoids)	Human iPSC	Kidney organoid–nephron progenitor cells and nephron epithelia
Freedman et al. 2015 [71]	CHIR and B27	16–23 days	Human iPSC	Kidney organoid–nephron segment cells
Ciampi et al. 2016 [72]	N2 and B27 supplements, CP21R7 (Roche), BMP4, retinoic acid, BMP7, FGF9, vitamin D3	13 days	Human iPSC	Podocyte-like cells
Musah et al. 2017 [73]	Activin A, CHIR, BMP7, VEGF, and retinoic acid	26 days	Human iPSC	Podocyte-like cells
Taguchi & Nishinakamura 2017 [74]	Activin, Bmp4, CHIR, FGF9, FGF1, GDNF, LDN193189, SB431542, retinoic acid, and B27	12.5 days	Human iPSC	Ureteric bud-like cells
Wu et al. 2018 [75]	CHIR, FGF9, heparin, Noggin, activin, and NTRK2 inhibitor K252a	25–26 days	Human iPSC	Kidney organoid–nephron progenitor cells
Przepiorski et al. 2018 [76]	CHIR and KnockOut Serum Replacement (KOSR)	14–26 days	Human iPSC	Kidney organoid–nephron progenitor cells
Rauch et al. 2018 [77]	Activin A, BMP7, and retinoic acid	10 days	Human iPSC	Podocyte-like cells
Mae et al. 2018 [78]	Activin A, CHIR, BMP4, LDN193189, A83-01, retinoic acid, PD0325901, FGF8, TTNPB, GDNF, FGF1, thiazovivin	15 days	Human iPSC	Wolffian duct cells
Qian et al. 2019 [79]	CHIR and B27	16 days	Human iPSC	Podocyte-like cells
Hariharan et al. 2019 [80]	Activin A, BMP4, retinoic acid, GDNF, HGF, REGM, FGF2, and BMP7	6 - 14 days	Human iPSC	Renal progenitor cell–multiple nephronal cell
Ahmadi et al. 2019 [81]	CHIR, PD032590, activin A, TTNPB, BMP7, LIF, GDNF, retinoic acid, vitamin D3, dexamethasone	22 days	Mouse iPSC	Podocyte-like cells
Garreta et al. 2019 [82]	CHIR, FGF9, heparin, activin A	21 days	Human iPSC	Kidney organoid–nephron segment cells

**Table 2:** Published studies involving iPSC-derived renal cells in kidney diseases.

Authors	iPSC-derived cell type	Kidney disease type
Imberti et al. 2015 [64]	Renal progenitor cell	Cisplatin-induced AKI
Toyohara et al. 2015 [65]	Renal progenitor cell	Ischemia/reperfusion-induced AKI
Li et al. 2015 [66]	Renal progenitor cell	Ischemia/reperfusion-induced AKI
Hoshina et al. 2018 [83]	Renal progenitor cell	Ischemia/reperfusion-induced AKI
Ahmadi et al. 2019 [81]	Podocytes	Membranous nephropathy

## Data Availability

The data supporting this review are from previously reported studies and datasets, which have been cited in the manuscript.
